# Hemostasis and ageing

**DOI:** 10.1186/1742-4933-5-12

**Published:** 2008-10-23

**Authors:** Daniela Mari, Giulia Ogliari, Davide Castaldi, Giovanni Vitale, Elisa Mariadele Bollini, Domenico Lio

**Affiliations:** 1 Dipartimento di Scienze Mediche e U.O. di Geriatria, IRCCS, Istituto Auxologico Italiano, Università di Milano, Milano, Italy; 2 Dipartimento di Biopatologia e Metodologie Biomediche, Università di Palermo, Palermo, Italy

## Abstract

On March 19, 2008 a Symposium on Pathophysiology of Ageing and Age-Related Diseases was held in Palermo, Italy. The lecture of D. Mari on Hemostasis and ageing is summarized herein. Physiological ageing is associated with increased plasma levels of many proteins of blood coagulation together with fibrinolysis impairment. This may be of great concern in view of the known association between vascular and thromboembolic diseases and ageing. On the other hand, centenarians are characterized by a state of hypercoagulability and possession of several high-risk alleles and well-known atherothrombotic risk markers but this appears to be compatible with longevity and/or health. Parameters considered risk factors for atherosclerotic vascular diseases in young people may lose their biological significance in advanced age and assume a different role.

## Background

On March 19, 2008 a Symposium on Pathophysiology of Ageing and Age-Related Diseases was held in Palermo, Italy. The lecture of D. Mari on Hemostasis and ageing is summarized herein. Physiological ageing is associated with increased plasma levels of many proteins of blood coagulation, with fibrinolysis impairment. This may be of great concern in view of the known association between vascular and thromboembolic diseases and ageing.

### Pro-thrombotic clotting factors

The plasma concentrations of several clotting factors, namely fibrinogen, factor VII, factor VIII, von Willebrand factor (VWF), factor IX, factor XII, high molecular-weight kininogen, and prekallikrein increase with progressing age in healthy [1]. Meade et al [2] had shown in a population study of subjects, aged 53–64 years, significantly higher levels of fibrinogen (300 mg/dL) than those found in younger subjects aged 20 (250 mg/dL). As a whole, an increment of plasma fibrinogen level by 10 mg/dL for each decade can be expected in healthy subjects. The contribution of fibrinogen to cause thrombosis is not fully elucidated. Fibrinogen, moreover, is a molecule that plays a role in acute-phase inflammation and fibrinogen levels increase in response to interleukin-6 and both are strongly correlated with ageing [3]. Factor VII which is not an acute phase reactant, triggers the coagulation cascade when circulating factor VII reacts with tissue factor that is usually not expressed in the intact vasculature. Factor VII plasma levels progressively increase with age, from a mean of 95 units/dL in subjects of 20 years old to over 110 units/dL in subjects over 50. Thrombotic disorders have been shown to be more frequent in subjects with higher plasma levels of factor VII but the data have not been confirmed in other studies [4]. Unfortunately there are differences in factor VII coagulant assay methodologies. Factor VIII, another acute phase protein, acting as a cofactor in the activation of factor X promoted by factor IXa, progressively increases with age, reaching a mean of over 200 units/dL in the healthy subjects over sixty. The study of variation of the concentration of the active products of the coagulation factors (not of the concentration of the single factor, i.e. the hypercoagulability) is very important to investigate the relationship between haemostasis and ageing. With advancing age many individuals, who are otherwise normal, show laboratory evidence of heightened coagulation enzyme activity, i.e., hypercoagulability, as detected by elevated plasma levels of prothrombin fragment 1 +2 (F1+2), Fibrinopeptide A (FpA), Thrombin-antithrombin complex (TAT) and D-dimer (D-D). Prothrombin fragment 1 +2 is a measure of the cleavage of prothrombin by activated factor X (factor Xa) being released from the amino terminal portion of the molecule during its conversion to thrombin. FpA is a measure of the formation of fibrin being released from the α-chain of fibrinogen when thrombin converts fibrinogen to fibrin. TAT is a measure of thrombin generation and neutralization by antithrombin. DD is a stable degradation product of cross linked fibrin, a marker of endogenous fibrinolysis. The levels of factor IX and factor X activation peptides also increase with advancing age [5].

### Physiological inhibitors of blood coagulation

The main physiological inhibitors of blood coagulation are *natural anticoagulants *produced by the liver and circulating in the plasma, antithrombin III, heparin cofactor II, the protein C-protein S system, and tissue factor pathway inhibitor (TFPI). The increased activation of the age-related coagulation is not due to a decrease in the main inhibitor of the tissue factor pathway, TFPI, which increases with ageing. The behaviour pattern of TFPI is gender dependent; in women, statistically significant increases in the plasma concentration of TFPI with age have been observed, paralleling the rise in factor VII. No significant age-related change in TFPI has been found in men [6]. Protein C levels are normal in the elderly and levels of protein C activation peptides are increased as well with age [5], excluding that a reduced activity of the endothelial thrombomodulin can trigger the activation of the coagulation. Hypercoagulability in apparently normal males of increasing age is not supported by the decreased functional activity of the antithrombin III per se. The occurrence of menopause significant increases antithrombin III levels, but the increased generation of activated factor X could depend on a minor efficiency of the inhibitory system of ATIII, due to a deficient displacement of the endothelial glycoasaminoglycans modulating the ATIII activity *in vivo *[1]. As with other epidemiological studies, Lowe et al [7] in the course of the Third Glasgow MONICA Survey observed that the median antithrombin levels decreased in male elderly, but increased after menopause in women before falling again at an older age. The plasma concentration of free protein S increased with ageing; in the healthy blood donors free protein S levels rose from 86% in subjects under 40 years to 99% in subjects over 40 years of age. Recently data from the Cardiovascular Health Study have assessed the associations of common polymorphisms of protein C (PROC), protein S (PROS1) and soluble plasma protein C receptor (sEPCR) and plasma protein C, soluble protein C receptor and protein S levels measured in a sub-sample of 336 participants at study entry [mean age 77 ± 7.4 (65–98), 47% female]. The results of this study showed that the PROCR Ser219Gly polymorphism (rs867186) was strongly associated with higher sEPCR levels, explaining 75% of the phenotypic variation. The Ser219Gly variant was also associated with higher levels of circulating protein C antigen. The minor alleles of PROC rs2069901 and PROS1 rs4857343 were weakly associated with lower protein C and free protein S levels, respectively. There was no association between PROCR Ser219Gly and risk of coronary heat disease, stroke, or mortality. The minor allele of another common PROCR tagSNP, rs2069948, was associated with lymphoid PROCR mRNA expression and with increased risk of incident stroke and all-cause mortality, and decreased healthy survival during follow-up [8]. Heparin cofactor II (HCII), a serine protease inhibitor (serpin), inhibits thrombin actions after binding to dermatan sulphate at injured arterial walls and may negatively regulate thrombin actions in vascular walls. With the use of ultrasound imaging of the carotid artery, a study performed in 306 Japanese elderly individuals (154 men and 152 women; age, 40 to 91 years; 68.9 +/- 11.1 years, mean +/- SD) demonstrated that plasma HCII activity decreased with age and negatively correlates with the severity of carotid atherosclerosis. Then the HCII may be identified as a novel independent protective factor against carotid atherosclerosis [9].

### FV Leiden

Activated protein C is the underlying cause of the most prevalent inherited thrombophilia in individuals of European descent. The majority of the patients carry a single point mutation in the gene encoding factor V, an important protein in the clotting coagulation system and carriers of the mutation are at increased risk for venous thromboembolism [VTE], pregnancy complications, cerebral vein thrombosis, and possibly early myocardial infarction with normal coronary anatomy. Based on the increased risk of VTE events in persons with the FV Leiden gene, one might assume that persons who carry the FV Leiden mutation have a shortened life expectancy compared with those without the mutation. Studies on octogenarians and centenarians in Europe, however, suggest a normal life span for FV Leiden carriers. In 3 studies of people older than 80 years old living in Italy [10], Denmark [11] and the United Kingdom [12] the prevalence of the FV Leiden gene in the elderly population was similar to the prevalence of the FV Leiden gene in the general population. If FV Leiden carriers were dying at a younger age from VTE events, then the expected prevalence of the FV Leiden gene in an older age group should be lower. In a population-based mortality study of octogenarians living in Leiden, 660 patients were observed for 10 years. The relative risk of mortality in FV Leiden carriers was identical to that in no carriers [13].

### Fibrinolyis

Fibrinolysis activity is impaired in the elderly, particularly due to an increase in plasminogen activator inhibitor 1 (PAI-1) levels. The synthesis of PAI-1 is increased in activated or injured endothelial cells and smooth muscle cells, and abundant PAI-1 is also secreted by activated platelets. The increased expression of this potent inhibitor *in vivo *will suppress the normal fibrinolytic system and create a prothrombotic state, resulting in pathological fibrin deposition followed by tissue damage. Fibrinolytic activity decreased significantly in the middle-aged group as shown by a prolongation of the ECLT (P < 0.01) and PAI-1, although not significantly, increased by approximately 100%, whereas tissue plasminogen activator (t-PA) significantly increased in the middle-aged group (P < 0.01) [14]. In aged subjects plasma PAI-1 levels correlated with the degree of insulin resistance (r = 0.61, P < 0.001), fasting plasma triglycerides (r = 0.74, P < 0.001) and age (r = 0.33, P < 0.001) [15]. Increases in PAI-1 have also been observed in senescent human umbilical vein endothelial cells. Genotypes for PAI-1-675 4G/5G appeared to be associated with lower non-cardiovascular mortality in men, but with greater cardiovascular mortality in women in the Cardiovascular Health Study [16].

Thrombin Activatable Fibrinolysis Inhibitor (TAFI) is a 228 plasma carboxypeptidase that regulates fibrinolysis by removing the C-terminal lysine and arginine residues from fibrin, that are required for efficient plasmin formation. Cardiovascular Health Study emphasized the role of TAFI 438 A/A genotype in predicting mortality in all cases, which is decreased in white men followed up for 10 years, the mean age at study entry was 73 years. The additional years of life are 0.9 (95% CI 02–1.9 years) or 1.1 additional years of quality of life (95% CI 02–2.1 years), more than men with the 438G allele. No significant statistically effects are associated to TAFI 438 G/A in women. TAFI when activated can cleave several substrates and participates in the regulation of inflammation [16]. This TAFI 438 A/A genotype may have provided our ancestors with enhanced anti-inflammatory and antithrombotic mechanisms, to improve wound healing and facilitate reproduction. TAFI may be one of the genes selected because they confer a reproductive advantage early in life and may have harmful effects in the post-reproductive period; negative selection against these harmful effects fails because the force of natural selection declines with age [17].

### The centenarians

During the last century, preventive medicine and better living standard in the early stage of life have ameliorated responses to short-term infections, with delayed adverse effects during ageing. Mari et al [18] were the first to measure parameters of coagulation and fibrinolysis in *centenarians *and in older [51–69 years old] and younger controls [18–50 years old]. Older controls had slightly higher values of several coagulation and fibrinolysis measurement than younger controls. Centenarians have a state of hypercoagulability with striking signs of high coagulation enzyme activity, as assessed directly by measuring factor VIIa in plasma (p < 0.01, compared with either control group) or indirectly by measuring the peptides released following the activation of prothrombin, or factor IX, or factor X (all p < 0.001). In addition, thrombin activity and enhanced formation of fibrin is evidenced by high circulating levels of fibrinopeptide A (p > 0.001) and secondary high hyperfibrinolysis, as reflected by elevated levels of D-dimer and of plasmin-antiplasmin complexes (both > 0.001). VWF, a well-known atherothrombotic risk marker is extremely high in centenarians. Fifty-one percent of centenarians have a reduction of the relative proportion of high molecular weight multimers [HMW]; furthermore VWF-cleaving protease was lower than in young controls [19]. Centenarians have anti-aCL and anti-beta-2GPI antibodies with a pattern comparable to that present in sera from patients suffering from the antiphospholipid syndrome. In spite of the presence of antibodies comparable to those found in patients with the anti-phospholipids syndrome, no vascular events were reported suggesting the presence of unknown protective factors and/or the lack of triggering factors [20]. The study on Italian centenarians suggests that the 4G allele carriers reached longevity despite high circulating PAI-1 level. Centenarians have a paradoxically significantly higher frequency than young individuals of the high-risk genetic markers mutant factor V (Arg506Gln) and prothrombin gene G20210A mutation [21,22]. Thus, the state of hypercoagulability and the possession of several high-risk alleles and well-known atherothrombotic risk markers appear to be compatible with longevity and/or health.

## Conclusion

In the oldest old, the cardiovascular risk factors could play a different role that in young-adult subjects: for example, high total cholesterol concentrations are associated with longevity owing to lower mortality from cancer and infection [23]. Candore et al [24] demonstrated the different role of some proinflammatory alleles, such as pyrin and CCR5, in acute myocardial infarction and longevity. The results support the hypothesis that the genetic background favouring cardiovascular diseases is detrimental to longevity. Data about the hemostasis profile of centenarians once again emphasized the need for a multidisciplinary approach if we wish to understand the mechanisms of successful ageing and to establish the contribution of unknown protective genes.

## Competing interests

The authors declare that they have no competing interests.

## Authors' contributions

All authors contributed equally to the paper and read and approved the final manuscript.
